# Effects of Mindfulness-Based Positive Behavior Support (MBPBS) Training Are Equally Beneficial for Mothers and Their Children With Autism Spectrum Disorder or With Intellectual Disabilities

**DOI:** 10.3389/fpsyg.2019.00385

**Published:** 2019-03-06

**Authors:** Nirbhay N. Singh, Giulio E. Lancioni, Bryan T. Karazsia, Rachel E. Myers, Yoon-Suk Hwang, Bhikkhu Anālayo

**Affiliations:** ^1^Medical College of Georgia, Augusta University, Augusta, GA, United States; ^2^Department of Neuroscience and Sense Organs, University of Bari, Bari, Italy; ^3^Department of Psychology, The College of Wooster, Wooster, OH, United States; ^4^WellStar School of Nursing, Kennesaw State University, Kennesaw, GA, United States; ^5^Institute For Learning Sciences and Teacher Education, Australian Catholic University, Brisbane, QLD, Australia; ^6^Numata Center for Buddhist Studies, University of Hamburg, Hamburg, Germany

**Keywords:** Mindfulness-Based Positive Behavior Support, MBPBS program, autism spectrum disorder, perceived psychological stress, aggression, disruptive behavior, compliance

## Abstract

Parenting a child with autism spectrum disorder (ASD) or intellectual disabilities (IDs) can be stressful for many parents. Mindfulness-Based Positive Behavior Support (MBPBS) is a customized mindfulness program that enables parents and other caregivers to reduce their perceived psychological stress to normative levels through mindfulness procedures and to support children with ASD or ID to self-manage their challenging behaviors through positive behavior support (PBS). In this study, we evaluated whether MBPBS would have differential effects on the stress levels of mothers of adolescents with ASD (*n* = 47) or with ID (*n* = 45) and the effects of the program on the aggressive, disruptive, and compliance behaviors of their children. Both groups of mothers participated in the 40-week study (10 weeks control and 30 weeks MBPBS program), rated their own stress levels, and collected daily observational data on the adolescents’ behavior. Results showed significant reductions in the level of stress in both groups of mothers, but no differential effects on mothers of children with ASD or with ID. In addition, significant reductions in aggression and disruptive behavior and increases in compliance behaviors were observed in the adolescents in both groups. The results suggest that MBPBS is equally beneficial for mothers of adolescents with ASD or ID. In the present study, although the mothers of children with ID had slightly higher levels of stress at baseline and mothers of children with ASD had lower levels of stress following the MBPBS program, the program can be considered equally effective in reducing the stress levels of both groups of mothers. This suggests that the program may be effective regardless of baseline levels of mothers’ stress.

## Introduction

Situational stress can help people to cope with and engage in adaptive responses to adverse situations (de Kloet et al., 2005; Joels and Baram, 2009), but prolonged stress usually has serious negative effects on brain function and behavior (Lupien et al., 2009; McEwen, 2012). Parents of children with autism spectrum disorder (ASD) and intellectual disabilities (IDs) are at risk for prolonged stress because of a number of child characteristics, such as age of the child, severity of diagnostic condition, level of functional abilities, and especially behavioral challenges (Davis and Carter, 2008; Osborne and Reed, 2010; Estes et al., 2013; Lovell and Wetherell, 2016). In addition to dealing with the child’s chronic and periodically escalating behavioral challenges, parents may also have distal concerns regarding the child’s long-term welfare that exacerbate their stress (Hsiao, 2018). Parental stress negatively affects not only child caregiving, such as harsh parenting (Mortensen and Barnett, 2015) and intervention outcomes (Shine and Perry, 2010), but also parental mental and physical health (Miodrag and Hodapp, 2010; Lai et al., 2015). Furthermore, parental stress differentially affects outcomes for parents and children with ASD when compared to those with ID alone (Estes et al., 2009; Griffith et al., 2010).

A number of parenting programs, including the so-called third-generation of cognitive-behavioral approaches, have focused on ameliorating the stresses and strains of parenting children and adolescents with intellectual and developmental disabilities (McIntyre and Neece, 2016). The third-generation or third-wave of cognitive-behavioral approaches have typically focused on assembling and testing the effects of multi-component therapeutic *procedures* that target multiple treatment goals based on a number of variables, including therapeutic priority, immediacy of treatment needs, difficulty of the treatment, and other outcome dimensions specific to an individual (Hoffman and Hayes, 2018). While this approach has produced evidence-based interventions that enhance quality of care, current research is shifting toward developing broad-based multi-level programs that focus on therapeutic *processes* derived from theory-based, testable, mechanisms of change as the basis for new interventions to achieve short- and long-term goals of the individual (Hayes and Hofmann, 2018). Of the currently available therapies, mindfulness programs seem to align well with a therapeutic process approach because they work at multiple levels to produce short-term therapeutic change, as well as longer term transformational change in the individual.

The third-generation therapies currently used with parents of children with ASD or ID include approaches based on Acceptance and Commitment Therapy (ACT) and mindfulness-based (MB) programs (Duncan et al., 2009; Cohen and Semple, 2010; Whittingham, 2014). For example, Blackledge and Hayes (2006) provided a 2-day training in ACT to parents of children with autism using a within-subject design, with assessments undertaken twice before the training and twice following the training. Results showed significant reductions in general distress and depression levels, and reduced experiential avoidance and cognitive fusion that were maintained for 3 months following the training. In a small qualitative study, Reid et al. (2016) provided two 4-h workshops that included five key ACT concepts, “(i) stress is normal, (ii) how we use language and thoughts to problem solve, (iii) the downside to living in our thoughts, (iv) alternatives to living in our thoughts, and (v) being led by our values not by our thoughts.” (p. 7). Parents reported being able to better cope with stress and with generally positive effects on their own well-being as well as that of their children. These studies indicate that using ACT procedures enhance psychological flexibility—a key outcome of ACT—which is indicative of parents being able to use mindfulness skills. Although only exploratory, these studies suggest that ACT procedures may have beneficial effects on parents of children with developmental disabilities. It remains to be seen if randomized controlled trials (RCTs) of ACT show significantly reduced stress in such parents and reduced behavioral challenges in their children.

Research using MB practices is farther along than for ACT. For example, Neece (2014) evaluated the effectiveness of the standard Mindfulness-Based Stress Reduction program (MBSR; Kabat-Zinn, 1990) in a waitlist control design study. When compared to waitlist control parents and their children, outcomes of the MBSR training included significant reductions in parental stress and depression, and reduction in behavior problems of their children. In a large RCT, Dykens et al. (2014) evaluated the comparative effects of MBSR and Positive Adult Development programs in a sample of 243 mothers of children with autism or other disabilities. Although significant effects were noted in both groups on measures of stress, anxiety, depression, sleep, and well-being, mothers in the MBSR group showed greater positive changes than the Positive Adult Development group on all variables except for stress. In a feasibility study, Lunsky et al. (2015) assessed the effects of a customized 6-week MB coping with stress group training with parents of adolescents and adults with developmental disabilities. Although the study was uncontrolled, initial results indicated the MB group training resulted in significant reduction in parental stress, but had no effect on their mindfulness or mindful parenting scores. Bazzano et al. (2015) adapted the standard MBSR program to address parent and professional caregiver stress. In a quasi-experimental design study, they tested the effects of this 8-week program in community settings, with training provided in both English and Spanish. Results indicated a significant reduction (by 33%) in the parents’ and caregivers’ perceived stress, and a 22% reduction in parental stress. In addition, based on self-report measures, both groups of participants reported significantly increased mindfulness and well-being. The positive effects were maintained at the 2-month follow-up assessment.

As with ACT programs, research on MBSR and adapted MBSR programs for parents of children (including adolescents and adults) with developmental disabilities show that parents can learn skills that reduce their stress levels, but they do not teach them how to better manage their children’s challenging behavior—ostensibly the source of much of their stress. It is likely that escalating behavioral challenges evidenced by their children will test the parents’ newly acquired stress management skills and they may likely revert to high levels of chronic stress. Positive Behavior Support (PBS) is an evidence-based approach that parents and professional caregivers use to support children, adolescents, and adults who engage in challenging behaviors, such as anger, aggression, and disruptive behavior (MacDonald, 2016; Morris and Horner, 2016). Combining MB training with PBS braids two evidence-based practices—Mindfulness-Based Positive Behavior Support (MBPBS)—that can be taught to parents and other caregivers and seamlessly implemented in multiple contexts, such as family home, group homes, large residential centers, schools, and other community-based settings (Singh et al., 2016c). The mindfulness component of the MBPBS program was designed to include a broad array of meditations that go beyond the traditional secular MB programs. The reasoning behind this approach was that the program needs to be focused not only on enhancing the psychological and emotional well-being of the parents and their children, but also on putting them on a pathway to personal transformation and transcendence (Vago and Silbersweig, 2012).

The findings from initial implementation of MBPBS by caregivers of individuals with developmental disabilities indicated that the combined procedure was effective in significantly reducing caregiver stress and in managing the aggressive behavior of individuals with developmental disabilities in their care (Singh et al., 2015, 2016b). Furthermore, findings from these studies were confirmed in two subsequent RCTs (Singh et al., 2016a, 2018). Indeed, the early MB parent training studies that provided the basis for the development of MBPBS showed that the effects of parental mindfulness training cascades to the behavior of their children who did not receive mindfulness training (Hwang and Singh, 2016; Singh et al., 2017). For example, parental training in mindfulness decreased aggression, non-compliance and self-injury in children with autism (Singh et al., 2006), and decreased aggression and increased social behavior in children with developmental disabilities (Singh et al., 2007). In a proof-of-concept study, mothers of adolescents with ASD attended a MBPBS training program for 1 day a week for 8 weeks in a 48-week study. The training resulted in statistically significant reductions in the mothers’ stress levels. In addition, the adolescents’ challenging behaviors decreased and compliance with parental requests increased. In sum, there is growing evidence that training in MBPBS enables mothers and other caregivers to reduce their stress levels to within “normal” levels, and they are able to support individuals with ASD and ID to significantly reduce their challenging behaviors and increase socially acceptable behaviors.

The issue of providing effective training to parents of children with ASD is critical because of the growing prevalence of the disorder when compared to those with related disorders. For example the prevalence of ID has remained steady over the last decade at about 1.04% or 10.37 per 1,000 individuals (Maulik et al., 2011; McKenzie et al., 2016). The prevalence of autism was estimated at 0.5 per 1,000 in the 1960s, but it has increased dramatically in the last decade (Newschaffer et al., 2007). The average estimate of current prevalence of ASD is 16.8 per 1,000 or 1 in 68 (range = 13.1 to 29.3 per 1,000 children aged 8 years) as of 2014 in the United States (Baio et al., 2018). Given the challenges associated with the disorder, it is not surprising that mothers of children with ASD experience greater levels of stress than mothers of children with ID alone (Estes et al., 2009; Griffith et al., 2010). Another consequence of having a child with ASD is the much higher risk for parental divorce when compared to parents with children that have other types of disabilities (Hartley et al., 2010). Furthermore, there is the added financial burden of caring for a child with ASD. Current estimates suggest that a child with ASD costs in the range of $4,110–6,200 more annually when compared to children without autism, with an annual national cost of $11.5–60.9 billion dollars (Center for Disease Control and Prevention [CDC], 2018).

Given these considerations, it is imperative that parents of children with ASD have access to programs that not only reduce their psychological stress, but also enhance their capability of positively caring for their children who exhibit challenging behaviors, such as aggression and disruptive behaviors. While several training programs with varying degrees of effectiveness are available (see McIntyre and Neece, 2016 for a review), there have been calls in the parenting literature that mothers of children with ASD may need additional interventions that incorporate “content specific to the challenges these parents and families face” (Cachia et al., 2016, p. 12) when compared to parents of children with ID. Conversely, research has not shown that the differences far outweigh similarities in parenting children with ASD or with ID alone to require interventions to be customized to each group of mothers. It could be that the differences may lie more in the degree than the nature of stressors affecting parents of children with ASD or with ID alone. Thus, the aim of this study was to evaluate whether training on MBPBS will have differential effects on mothers and their children with ASD or with ID alone in terms of the mothers’ stress levels and the children’s aggressive, disruptive, and compliance behaviors.

## Materials and Methods

### Ethics Statement

All training procedures in the study were in accord with the 1964 Helsinki declaration and its later amendments or comparable ethical standards (e.g., obtaining assent from the children and consent from their parents; Carlson et al., 2004). Written informed consent was obtained from all individual participants involved in the study. This study was carried out in accordance with the recommendations of American Psychological Association (APA) Ethical Principles of Psychologists and Code of Conduct with written informed consent from all subjects.

### Participants

The mothers of children with ASD or ID were recruited from the community, via flyers at medical centers, family physicians, and local service agencies, and by word-of-mouth over a period of 3 years and 5 months. Given the experimental design required equivalent diagnostic groups rather than random allocation of participants, recruitment continued until an equal number of eligible mothers were allocated to the two groups. A power analysis using the software G^∗^Power 3.1 indicated that 86 participants were needed to detect a medium effect size (*f* = 0.25) of a within-between interaction (time × group), between subject factors (i.e., group), and within subject factors (i.e., time) of a repeated measures ANOVA with 80% power at the 0.05 significance level (Faul et al., 2009). The expected effect size was based on earlier studies using MBPBS (e.g., Singh et al., 2014, 2016a, 2018) and reviews of mindful parenting (e.g., Cachia et al., 2016). We oversampled because of expected dropouts prior to and during the study.

**FIGURE 1:**
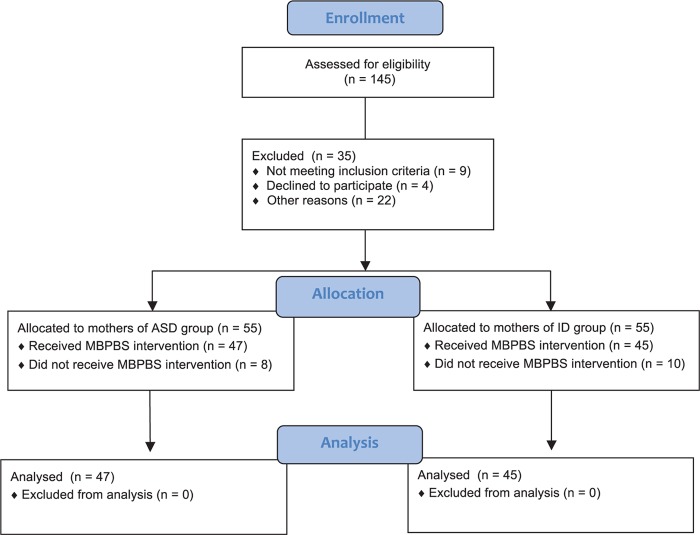
CONSORT flow diagram.

Mothers were included in the initial pool of possible participants if they had only one adolescent (aged between 13 and 19 years) with ASD or ID. A total of 145 mothers were assessed for eligibility and, of these, 35 were excluded because they did not meet eligibility criteria [e.g., did not meet inclusion criteria, declined to participate, other reasons (i.e., language barriers, training schedule conflicts, questionable diagnostic workup of their children, transportation issues)]. Of the 55 mothers allocated to the ASD group, eight dropped out for personal reasons prior to intervention and did not receive training in MBPBS. Of the 55 allocated to the ID group, 10 dropped out for personal reasons prior to training on MBPBS. Figure 1 presents a CONSORT flow diagram of participant allocation. On average, the mothers had one to three typically developing children, but only one child with ASD or ID diagnosis. Table 1 presents the characteristics of the mothers and their children with ASD or ID.

**Table 1:** Socio-demographic characteristics of the mothers and their adolescents with either autism spectrum disorder (ASD) or intellectual disabilities (IDs).

	ASD		ID	
		
	Mothers	Adolescents	Mothers	Adolescents
Number of participants	47	47	45	45
Mean age/years	47.91	15.15	48.82	15.56
Age range (years)	39–59	13–17	38–55	13–17
Sex: females	47 (100%)	16 (34%)	45 (100%)	15 (33%)
Level of functioning				
ASD Level 1	na	47 (100%)	na	na
Mild	na	na	na	45(100%)
Number of individuals on psychotropic medications	na	22 (46.81%)	na	21 (46.67%)
Number of individuals with mental illness	na	12 (25.53%)	na	21 (46.67%)
Number of individuals with behavior plans for aggressive behavior	na	24 (51.01%)	na	26 (57.78%)


*Note: na = not applicable.*

### Procedure

#### Experimental Design

We used an equivalent diagnostic two-group design, with a control and intervention condition. In a 40-week study, the first 10 weeks constituted a control phase during which no additional procedures were instituted, followed by a 3-day training in MBPBS for both groups, and 30 weeks of implementation. The training was the same for both groups.

#### Experimental Conditions

##### Control

The first 10 weeks of the study served as a control phase enabling within and between diagnostic group comparisons. The mothers of children with ASD or ID were not provided with additional instructions on how to manage their children and were reminded to continue with their current practices without alteration in any way. In addition, the mothers were to continue with their usual self-care practices for their own physical and mental health.

##### Training

The 3-day MBPBS protocol was used in the intervention phase (see Table 2 for details of daily sessions). This training protocol is based on the standard 7-day MBPBS course (see Singh et al., 2018), but reduced in terms of the required time commitment by the parents. The course was presented, in small groups in the community, in three parts during three consecutive days. The first day was devoted to training and practice in mindfulness procedures that included three basic meditations, four immeasurables, five hindrances, three poisons, beginner’s mind, and ethical precepts. It also covered issues related to home practice, daily logs of meditation, journaling, and data collection. The second day was devoted to developing positive behavior intervention and support (PBIS) plans. Training included the following components: guiding principles, goals for the mother and child, gathering PBIS plan-specific information, assessment instruments, designing PBIS plans, questions for designing PBIS plans, and data collection and reliability procedures. The third day was devoted to a review and practice of daily meditation practices for the mothers, day-to-day implementation of an integrated MB positive behavioral intervention with their child, living mindfully with their family on a daily basis, informal mindfulness practices, collection and interpretation of outcome data, and frequently asked questions.

**Table 2:** Outline of the 3-day MBPBS Program.

Day 1 (Mindfulness training)	Samatha, Kinhin, and Open Monitoring meditations
	Five hindrances—sensory desire, ill will, sloth and torpor, restlessness and remorse, and doubt
	Four Immeasurables (*Brahmavihara*: *metta*—lovingkindness; *karuna*—compassion; *mudita*—empathetic joy; *upekkha*—equanimity)
	The three poisons—attachment, anger, and ignorance
	Beginner’s mind
	Practicing ethical precepts—refrain from (a) harming living creatures, (b) taking that which is not given, and (c) incorrect or false speech
	Daily logs and journaling
Day 2 (PBS training)	Review of the meditation instructions and practices (daily logs)
	Practice Samatha, Kinhin, and Open Monitoring meditations
	Review of the five hindrances, four immeasurables, three poisons, beginner’s mind, and ethical precepts
	Developing positive behavior intervention and support (PBIS) plans
	Guiding principles
	Goals for mother and child
	Gathering PBIS plan-specific information
	Functional assessment tools
	Developing specific hypotheses
	Designing PBIS plans
	Function-based modifications
	Teaching alternative skills
	Changing the consequences
	Providing long-term supports to enhance quality of life
	Questions for designing PBIS plans
	Implementing the PBIS plans and data collection procedures
Day 3 (Mindfulness and PBS training and practice)	Review of the meditation instructions and practices (daily logs)
	Practice Samatha, Kinhin, and Open Monitoring meditations
	Review of the five hindrances, four immeasurables, three poisons, Beginner’s mind, and ethical precepts
	Review and practice developing PBIS plans
	Putting it all together as a seamless package of practices
	Implementing MBPBS practice


##### MBPBS implementation

Following the 3-day training, the mothers implemented the MBPBS procedures in daily life, including their own meditation practices and developing and implementing PBS plans for their children, as needed. The MBPBS implementation procedure was similar to that used by Singh et al. (2014) in the initial multiple-baseline proof-concept study with mothers of children with ASD. The mothers had regular contact with the research team on a weekly basis for clarification of any procedures, questions arising from their daily practice, and reporting on their daily meditation practice.

##### MBPBS trainer

The MBPBS trainer had a life-long practice of Buddhist meditation practices and over 30 years of practice as a meditation instructor. In addition, the trainer had extensive experience as a behavior analyst, certified at the BCBA-D level, and over 40 years of experience in developing and implementing behavior intervention plans.

##### Fidelity of MBPBS Training

The training was videotaped for 10–12 min per hour per day during random segments of each hour of training. Twenty-four videotaped segments were rated for fidelity of MBPBS training by two independent raters, one an expert in mindfulness and the other an expert in PBS. The fidelity of MBPBS training was rated at 100% for meditation instructions and for principles, components of PBIS plans, and applications of PBS.

#### Measures

##### Training attendance

We recorded the mothers’ attendance at the 3-day MBPBS training.

##### Meditation practice

The mothers recorded in their daily logs the total time they spent in meditation practice each day during the 40-week study.

##### Perceived Stress Scale

The 10-item Perceived Stress Scale (PSS-10; Cohen et al., 1983) is a self-report questionnaire that was used to provide a subjective evaluation of lack of control (e.g., *In the last month, how often have you felt that you were unable to control important things in your life?*), unpredictability (e.g., *In the last month, how often have you been upset because of something that happened unexpectedly?*), and overload in the mothers’ daily life (Cohen and Williamson, 1988). The mothers responded on a five-point Likert scale format that ranged from 0 (never) to 4 (very often). The total score is calculated by reverse coding four items (i.e., 4, 5, 7, and 8) and then adding the scores of all 10 items, with higher scores indicating greater levels of perceived stress. The scale has adequate psychometric properties, with Cronbach’s alpha of 0.78 (Cohen and Williamson, 1988) and 0.80 for the present study. The mothers completed PSS-10 three times: on the first day of the control phase (Week 1); last day of control phase (Week 10); and the last day of MBPBS implementation (Week 40).

##### Aggressive behavior

Aggressive behavior was defined as the adolescent biting, hitting, scratching, punching, kicking, and slapping directed at any nuclear family member, or destroying property. This definition includes the universe of specific acts defined as aggression, but individual adolescents could engage in a single behavioral act or different combinations of these behaviors. Aggressive behavior data were aggregated in terms of number per week for the 40 weeks of the study.

##### Disruptive behavior

Disruptive behavior was defined as acts of the adolescent that “negatively affected the family’s social interactions, including pushing, shoving, inordinate or inappropriate demands for time or attention, creating excessive noise, offensive verbal comments, performing distracting repetitive acts during social interactions, and other idiosyncratic behaviors identified by the mothers of the adolescents in this study” (Singh et al., 2014, p. 648). Disruptive behavior data were aggregated in terms of number per week for the 40 weeks of the study.

##### Compliance with mother’s requests

Compliance was defined as the adolescent “responding to his mother’s requests in a socially appropriate manner within an acceptable timeframe that was determined by each mother” (Singh et al., 2014, p. 643). Compliance data were aggregated in terms of mean percentage per week.

The mothers collected data on their adolescent’s behavior using an App that allowed recording of multiple events in real time. They collected data when the adolescent was at home and in their mother’s presence (i.e., excluding times when the adolescent was in the bedroom or during personal and private time). On average, the mothers collected data on adolescent behavior for about 5 h during weekdays and about 8 h during weekends.

To establish inter-rater agreement, fathers also collected data on their adolescents’ behaviors using the same system but independently of the mothers, for an average of 2 h each week, usually during evenings and weekends. Inter-rater agreement was defined as both parents recording an instance of a specific behavior at about the same time (i.e., within ± 5 s). We calculated percent inter-rater agreement for each week by dividing the total number of agreements by the total number of observations made during that period and multiplying by 100. The inter-rater agreement for all observations with both parents present ranged from 83 to 100%, with a mean of approximately 90%.

### Data Analyses

For observed aggressive behavior, observed disruptive behavior, overall compliance, and maternal meditation, the unit of analysis was a count variable (either number of instances or number of minutes) for all individuals within a specified group. We adopted a strategy employed previously (Singh et al., 2016a) in which these group-level data were analyzed in an *n* of 1 framework, with each group being considered a single unit. To accomplish this, we plotted each count variable for each group across all weeks of the study. To supplement the visual presentation, we computed a Phi coefficient with a corresponding *p*-value (Parker et al., 2007; Parker and Vannest, 2009). This coefficient represents the extent to which data from a control and an intervention phase overlap. If there is no change in behavior across phases, then data points will overlap completely (Phi = 0.00); if there is substantial change in behavior, then data points will not overlap at all (Phi = 1.00). The associated *p*-value represents the probability that the obtained results are due to chance. We also averaged counts per week across the 10-week control phase and the 30-week implementation phase for each group. As the unit of analysis was not individuals, we were not able to utilize a mixed-model ANOVA.

Data regarding perceived stress were reported at the individual level, and thus a mixed-model ANOVA was used to compare main effects of group, time, and their interaction. Effect sizes reported include η^2^ for an overall effect size (with 0.02, 0.15, and 0.35 being small, medium, and large, respectively), or for a specific condition across time.

## Results

### Demographic Variables

A series of chi-square and independent samples *t*-tests revealed that there were no significant differences between diagnostic groups with regard to age of participants (mothers or adolescents), biological sex, use of psychotropic medications, or presence of behavior plans (all *p* > 0.05; see Table 1).

### Mother Variables

#### Training Attendance

All mothers in the ASD and ID groups attended all 3 days of MBPBS training.

#### Meditation Practice

None of the mothers in either the ASD group or the ID group engaged in meditation during the control phase. On average, the mothers in the ASD and ID groups meditated for 18.77 and 18.23 min daily during the 30 weeks following the training, respectively.

#### Perceived Stress

**FIGURE 2:**
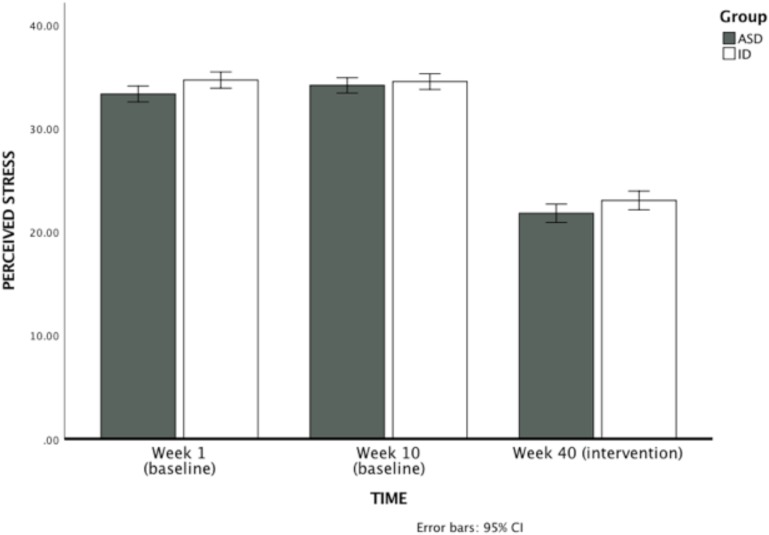
Mothers’ ratings of perceived psychological stress on PSS-10 during the first week of the control condition, the last week of the control condition (week 10), and the last week of MBPBS intervention (week 40). Higher scores indicate greater psychological stress.

A 2 (group: ASD versus ID) × 3 (time: control week 1, control week 10, MBPBS implementation week 40) mixed-model ANOVA was used to examine maternal reports of perceived stress across groups and across the three phases of assessment (control week 1, control week 10, and MBPBS intervention week 40). The between subjects factor was statistically significant, *F*(1,90) = 4.98, *p* = 0.028 (η^2^ = 0.052). This revealed an overall group effect, with mothers in the ID group reporting significantly higher stress (*M* = 30.72, *SD* = 1.79) than mothers in the ASD group (*M* = 29.74, *SD* = 2.37). There was also a significant effect of assessment time, *F*(2,89) = 637.82, *p* < 0.001 (η^2^ = 0.935), with no significant interaction effect *F*(2,89) = 2.21, *p* = 0.12 (η^2^ = 0.047). See Figure 2 for a visual depiction of these trends.

### Adolescent Variables

#### Autism Spectrum Disorder

**FIGURE 3:**
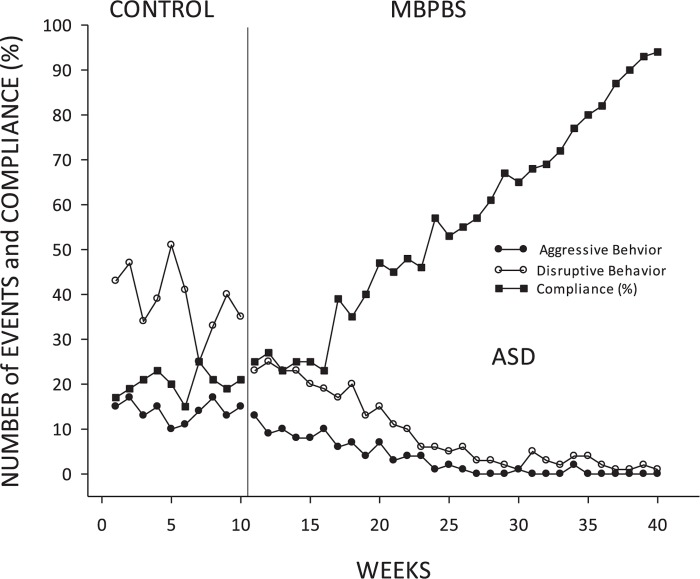
Adolescents with ASD: mean frequency of aggressive and disruptive behavior and mean percent of compliance with mother’s requests per week during each week of control and MBPBS phases.

Weekly counts of aggressive and disruptive behavior decreased substantially from the control phase to the intervention phase (aggressive behavior: phi = 0.71, *p* < 0.001; disruptive behavior: phi = 0.86, *p* < 0.001). Expressed as means and standard deviations across phases, aggressive behavior decreased from control (*M* = 14.00, *SD* = 2.31) to intervention (*M* = 3.33, *SD* = 3.92). Disruptive behavior also decreased from control (*M* = 38.80, *SD* = 7.47) to intervention (*M* = 9.20, *SD* = 8.25). Percent compliance increased substantially across these phases (phi = 0.86, *p* < 0.001), reflecting a change from control (*M* = 20.10, *SD* = 2.85) to intervention (*M* = 55.83, *SD* = 22.50). See Figure 3 for a visual depiction of these trends.

#### Intellectual Disabilities

**FIGURE 4:**
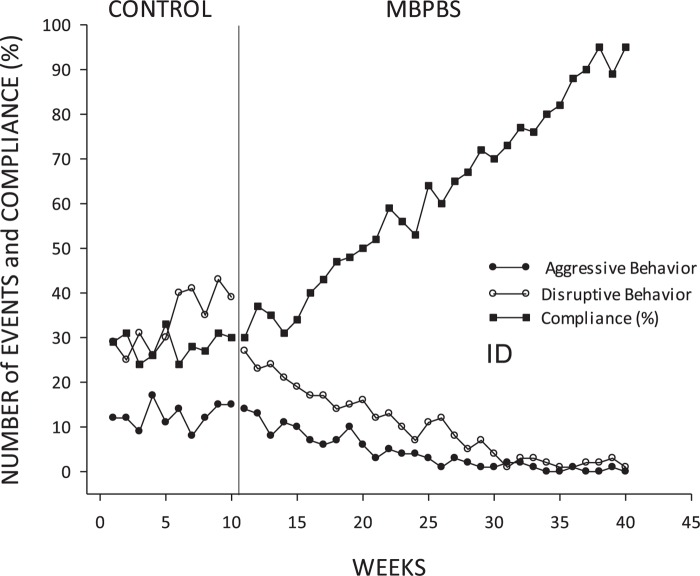
Adolescents with ID: mean frequency of aggressive and disruptive behavior and mean percent of compliance with mother’s requests per week during each week of control and MBPBS phases.

Among adolescents with ID weekly counts of aggressive and disruptive behavior decreased from the control phase to the intervention phase (aggressive behavior: phi = 0.66, *p* < 0.001; disruptive behavior: phi = 0.87, *p* < 0.001). In terms of means and standard deviations across phases, aggressive behavior decreased from control (*M* = 12.50, *SD* = 2.80) to intervention (*M* = 4.20, *SD* = 4.12). Disruptive behavior also decreased from control (*M* = 33.90, *SD* = 6.56) to intervention (*M* = 10.03, *SD* = 7.87). Percent compliance increased substantially across these phases (phi = 0.87, *p* < 0.001), reflecting a change from control (*M* = 28.30, *SD* = 3.06) to intervention (*M* = 61.93, *SD* = 20.00). See Figure 4 for a visual depiction of these trends.

## Discussion

Many mothers and other caregivers report elevated levels of chronic stress while raising or caring for children with ASD and ID. A small number of training programs based on the so-called third-generation of cognitive-behavioral approaches have been developed and evaluated, usually in non-RCT trials (Duncan et al., 2009; Cohen and Semple, 2010; Whittingham, 2014; Donnchadha, 2018). MBPBS is prototypic of these approaches and has been evaluated for its effectiveness and efficacy across a number of studies using single-subject, quasi-experimental, and RCT trials (Hwang and Singh, 2016; Singh et al., 2016c, 2017). In an effort to extend the findings of these studies, the present study investigated whether training in the 3-day MBPBS program would have differential effects on mothers and their children with ASD or ID.

As reflected in the eta-squared values, the total sample change across time accounted for far more variance (93.5%) than did the minor group effects (5.2% of variance explained). This indicates that the very small group differences were maintained throughout all phases of the study. In other words, the lack of an interaction effect indicates that both groups of mothers responded equally well to MBPBS, and response to the intervention accounted for far more variance in perceived stress than did group membership. In short, MBPBS did not have a differential effect on mothers of children with ASD or with ID. This finding can be interpreted in different ways. First, even though mothers of children with ASD have significantly more stress than mothers of children with ID, the similarities between the two groups of mothers outweigh their differences and thus do not require differential interventions based on the diagnosis of their children. Second, regardless of the differential stress levels of the two groups of mothers, the impact of the MBPBS program is strong enough to overcome the differences. We suspect, and this is a matter for future research, that the different stress levels is not inherent in the two groups of mothers but a result of cumulative effects of different presentations of challenging behaviors of children with ASD and with ID in terms of type and topography (e.g., stereotypy, rituals, aggression, destructive behaviors), severity, duration, intensity, and frequency. If this is the case, then the intervention would favor programs that teach the mothers how to reduce their stress levels and more skillfully support their children. MBPBS may be effective for both groups of mothers because it focuses on these two components. Of course, other approaches may have differential effects, but it does warrant examining whether different interventions are necessary for reducing stress in these two groups of mothers simply on the basis of their children’s diagnostic grouping.

Braiding mindfulness meditation practices with the practical behavior management strategies derived from PBS appear to enhance the mothers’ ability to not only effectively manage the behavior of their children, but also better manage their own psychological stress. It is likely that the breadth of mindfulness practices included in MBPBS enables mothers to re-perceive (Shapiro et al., 2006) their interactions with their children. That is, they learn to shift their attention away from the negative emotional arousal that results from their children’s challenging behaviors to their moment-by-moment interactions with objectivity and awareness, and to refrain from automatic reactivity. Re-perceiving may enable the mothers to lower the psychological distress that may arise from the challenging behaviors of their children (Harnett et al., 2016; Uusberg et al., 2016). This suggests that enhanced mindfulness may buffer the stressful consequences of negative mother–child interactions as postulated in the stress-buffering model of Cohen and Edwards (1989) and the disability-stress-coping model (Wallander et al., 1989). Future research may investigate whether the enhanced mindfulness mothers may derive from training in MBPBS attenuates the association between their children’s challenging behaviors and their own psychological stress.

Given the breadth of meditation practices encompassed in the MBPBS program, other mechanisms of change most likely came into play, including those that have been posited for mindfulness itself (Vago and Silbersweig, 2012; Bernstein et al., 2015; Carona et al., 2016). For example, compassion—defined as “sensitivity to suffering in self and others, with a deep commitment to try to relieve it” (Gilbert, 2010, p. 3)—is included in the MBPBS program. In Buddhist teachings, mindfulness and compassion are inextricably linked to awakening (Brach, 2004), but in programs such as MBPBS, mindfulness may aid mothers to see the reality of their situation as it is without bias, and compassion may provide them with the antidote and skillful means of overcoming their stress and related suffering (Wallace and Shapiro, 2006). The current paper was not designed to investigate the mechanisms by which the MBPBS may produce the documented changes, but we recognize that individual components may have overlapping and synergistic mechanisms that need to be investigated in future research.

Mindfulness-Based Positive Behavior Support appears to be a fairly robust intervention in terms of its effects on parents, professional caregivers, and teachers, and the individuals with ASD and ID in their care (Singh et al., 2017). Its effects have been tested using different components and varying number of days of training. Recent controlled trials have used the final iteration of components and taught in a 7-day format (e.g., Singh et al., 2016a, 2018). However, given the diversity in the parent population, it is more than likely that dosage of training will be a factor in outcomes for parents in any intervention, including MBPBS. Several MB interventions have been developed as multi-component packages, with the presumption that one-size-fits-all for producing beneficial effects across outcome variables. But, participants of MB programs come from different cultures, are of different ages, have differing needs, evidence a variety of spiritual, psychological, and mental health needs, and bring with them different causes and conditions that may directly or indirectly affect intervention outcomes. MBPBS was developed with these issues in mind. Thus, following the stepped-care model of intervention (Davison, 2000), the training dosage in the present study was reduced to 3 days, with the option of providing further training if deemed necessary. When compared to longer training versions, the stepped-care MBPBS model is least restrictive in terms of participant costs and personal inconvenience, which are important considerations for those already burdened with chronic stress. Of course, there still remains the issue of predicting the level of training needed by specific participants before a particular training is instituted, and the question of whether there is a need for an algorithm that may assist therapists and trainers to determine who may need further training or when training needs to be stepped up. These issues at the intersection of stepped care and personalized medicine are for future research.

In a study using MBPBS with mothers of children with ASD, Singh et al. (2014) noted a difference between the earlier and more recent MB interventions. They termed the earlier programs as “the first generation of MB approaches that were developed as secular interventions for ameliorating psychological and physical distress” (Singh et al., 2014, p. 655) and the emerging programs as “second generation of MB approaches” (p. 655). This dichotomy in MB programs has since been developed further (Van Gordon et al., 2015) and research on several second-generation programs has been published (see the Special Section on this topic in the journal *Mindfulness*, 2019). The first generation of MB programs was secular and designed to enhance psychological and emotional well-being and the second-generation programs are based on Buddhist teachings and precepts that not only enhance well-being but also encourage personal transformation and transcendence. MBPBS is a second-generation program because it includes not only some of the traditional components of MB programs, but also Buddhist concepts such as the three poisons, four immeasurables, five hindrances, and ethical precepts (Singh et al., 2013, 2014, 2018). These additional teachings may help participants to better experience the transformative effects of mindfulness as they develop wisdom, ethical conduct, and meditation practices (Bodhi, 2011).

One of these Buddhist concepts concerns the three poisons. This concept can be practically applied to enable a better understanding of the difference between skillful and unskillful intentions and reactions. This reflects a fundamental distinction in Buddhist thought between what is skillful or wholesome (*kusala*) and what is its opposite. This fundamental distinction informs Buddhist ethics and provides the underlying rationale for much of its meditational practices.

Applying this distinction may help mothers to be more mindful in their interactions with their children with ASD or ID. Skillful intentions toward their children arise from a mind that is free from the three poisons, which are greed, aversion, and delusion. Unskillful intentions arising from greed, aversion, and delusion may lead to *dukkha* (i.e., suffering, disease, or stress) for both themselves as well as their children. Understanding the causes and conditions of their intentions and actions may enable mothers to be more psychologically flexible with their children, thus reducing their own stress (Ruskin et al., 2013).

Mothers receive grounding in attitudinal or emotional modes of attending to their mental state through teachings on the four immeasurables (*brahmavihara*), which include: lovingkindness (*metta*), compassion (*karuna*), empathetic joy (*mudita*), and equanimity (*upekkha*). The fundamental quality of the four immeasurables is unconditional, as in unconditional lovingkindness, which makes them immeasurable. Through specific meditations, the mothers develop a wish, an aspiration, a resolve, and inspiration to transform their attitude toward all sentient beings. The ensuing attitudinal change process enables the mothers to gain insight into their own mental states, or the quality of their mind at any given time, as well as the purported workings of the minds of their children with ASD or IDD. For example, mothers gain an understanding that their children may not have developed skillful ways of dealing with their *dukkha*, and are thus responding with anger, aggression, and destructive behaviors. This understanding may lead the mothers to respond to their children with feelings of lovingkindness and compassion instead of reacting with harsh discipline. They may invoke an aspiration and resolve to act skillfully to reduce the children’s suffering and the causes of suffering. Cultivation of the four immeasurables changes mother–child interactions, resulting in reductions in both the challenging behaviors in the children and the psychological stress in the mothers (see Hoffman et al., 2013 for a review of the effects of lovingkindness and compassion meditation of psychological functions). While confirmatory research evidence is needed, we suspect that when the four immeasurables are coupled with other meditations included in MBPBS, synergistic transformations occur in the mothers and the children.

Similarly, a recognition of the presence of any of five mental conditions that are reckoned to be hindrances, which are sensual desire (*kamacchanda*), aversion (*thinamiddha*), sloth and torpor (*thînamiddha*), restlessness and worry (*uddhaccakukkucca*), and doubt (*vicikiccha*), may help mothers to respond more mindfully to their children. Employing this conceptual framework may help the mothers to detect when one of these detrimental conditions is present in their own mind, which by their nature lead to lack of clarity and unbalanced reactions. Based on such recognition, they can gradually learn to become aware of the conditions that result in the arising of a hindrance, how to overcome the arisen hindrance, and how to prevent the hindrance from arising again in future (Anālayo, 2003). In this way, based on applying traditional Buddhist instructions on the systematic cultivation of mindfulness in relation to the hindrances to their actual situation, mothers can use the concept of the five hindrances to check on the mental state of their minds. Such checking is not confined to formal meditation practice but also applicable to daily life. Based on increased awareness of their own mental condition with the help of this conceptual framework, they can learn to adjust their actions accordingly.

Another relevant Buddhist concept concerns the experience of feelings (*vedana*). Whereas the hindrances are the topic of what in the Buddhist traditions is considered the fourth establishment of mindfulness (*satipatthana*), feeling tone is the second establishment of mindfulness. Here the main task is to learn to recognize if experience in the present moment comes with a positive, negative, or neutral affective tone. Learning such recognition helps to realize the degree to which action and reaction take place under the usually not noticed influence of such feelings. When negative feelings arise during mother–child interactions, the mother is aware of the fact that such feelings are transient and clinging to them is stressful. Thus, she can learn to non-judgmentally observe and let go of the feelings as they arise, thus preempting the stress associated with clinging to them.

Mindfulness-Based Positive Behavior Support includes instruction in three ethical precepts as it may apply to mother–child interactions, i.e., to refrain from (a) harming living creatures, (b) taking that which is not given, and (c) false speech. These precepts are presented in a positive manner in terms of what mothers can do as opposed to what they should refrain from. For example, the ethical imperative of refraining from harming living creatures is taught within the context of using alternative positive practices instead of restrictive physical restraints that some parents resort to when stressed and emotionally exhausted (Allen et al., 2006; Lecavalier et al., 2006). Similarly, false speech is taught within the context of harsh verbal discipline that may prove to be emotionally crippling to the child. In more general terms, MBPBS emphasizes the difference between the mechanistic functions of paying attention in the present moment and genuine mindfulness (*samma sati*) that always incorporates an element of moral sensitivity, an ethical dimension that differentiates actions that may be harmful or beneficial.

As is common in MB interventions, the present study used mothers’ self-report of perceived psychological stress as the marker of internal change. Often this assessment is paired with other outcomes from self-report rating scales, such as changes in the level of mindfulness and other internal states before and after a MB intervention. Undoubtedly, these measures provide a reasonably good indication of the effects of intervention but this method of ascertaining outcomes suffers from common method bias (Podsakoff et al., 2003). Biological markers have emerged as a more reliable and objective method of measuring subjective states and could be used to provide confirmatory evidence. For example, Ruiz-Robledillo et al. (2015) used cortisol awakening response and afternoon cortisol levels as biomarkers of subjective change in a pilot study of the effects of MB intervention on mood disturbance and health complaints of parents of children with ASD. Future research could measure changes in biomarkers that have been linked to acute and chronic stress, such as stress hormones [i.e., cortisol and adrenocorticotropic hormone (ACTH)] and markers of inflammation [i.e., tumor necrosis factor-alpha (TNF-alpha), and IL-6) in the bloodstream, that are being used in other areas of mindfulness research (e.g., Hoge et al., 2018). Including biomarkers could enable researchers to determine if the participants are experiencing a homeostatic or a more stressful state.

To preempt common method bias in the children’s data, we used mothers’ behavioral observations in real time instead of the more commonly used parent ratings (e.g., Aberrant Behavior Checklist, 2017; Aman and Singh, 2017). In addition, we included fathers as observers of their children’s behavior during the weekends at the same time as but independently of the mothers. This enabled the assessment of inter-rater agreement between the parents, showing that the reliability of the primary observer (the mother) exceeded the standard criterion of 80% inter-rater agreement (Ledford and Gast, 2018).

Given the growing evidence-base for the effectiveness of MB interventions for stress across different populations, perhaps it is time for our field to consider how to translate research to practice. For example, if other researchers can replicate the current findings on the effectiveness of MBPBS, it may be an indication that translational research on this intervention is warranted. If our evidence-based interventions cannot be translated into reducing *dukkha* of those who need it, then the best evidence supporting their use is of little practical consequence in the lives of people. How to make such interventions available to parents and other caregivers who need it, but do not have access to it, may be the next step in future research.

## Author Contributions

NS designed and executed the study, assisted with data analyses, and wrote the first draft. GL collaborated on the design and writing of the study. BK analyzed the data and drafted the section “Results.” RM and Y-SH assisted with the writing, editing, and revision of the manuscript. BA assisted with the finer points of Buddhist teachings from an Early Buddhist perspective.

## Conflict of Interest Statement

The authors declare that the research was conducted in the absence of any commercial or financial relationships that could be construed as a potential conflict of interest.

## References

[B1] Allen D. Hawkins S. Cooper V. (2006). Parents’ use of physical interventions in the management of their children’s severe challenging behavior. *J. Appl. Res. Intel. Disabil.* 19 356–363. 10.1111/j.1468-3148.2006.00292.x

[B2] Aman M. G. Singh N. N. (2017). *ABC-2: Aberrant Behavior Checklist* 2nd ed. East Aurora, NY: Slosson.

[B3] Anālayo B. (2003). *Satipatthāna: The Direct Path to Realization.* Birmingham: Windhorse Publications.

[B4] Baio J. Wiggins L. Christensen D. L. Maenner M. J. Daniels J. Warren Z. (2018). Prevalence of autism spectrum disorder among children aged 8 years—Autism developmental disabilities monitoring netwek, 11 sites, united states, 2014. *MMWR Surveil. Summar.* 67 1–23. 10.15585/mmwr.ss6706a1 29701730 PMC5919599

[B5] Bazzano A. Wolfe C. Zylovska L. Wang S. Schuster E. Barrett C. (2015). Mindfulness-based stress reduction (MBSR) for parents and caregivers of individuals with developmental disabilities: a community-based approach. *J. Child Fam. Stud.* 24 298–308. 10.1007/s10826-013-9836-9

[B6] Bernstein A. Hadash Y. Lichtash Y. Tanay G. Shepherd K. Fresco D. M. (2015). Decentering and related constructs: a critical review and meta-cognitive processes model. *Pers. Psychol. Sci.* 10 599–617. 10.1177/1745691615594577 26385999 PMC5103165

[B7] Blackledge J. T. Hayes S. C. (2006). Using acceptance and commitment training in the support of parents of children diagnosed with autism. *Child Fam. Behav. Ther.* 28 1–18. 10.1300/J019v28n01_01

[B8] Bodhi B. (2011). What does mindfulness really mean? A canonical perspective. *Contemp. Buddhi.* 12 19–39.

[B9] Brach T. (2004). *Radical Acceptance: Embracing Your Life With the Heart of a Buddha.* New York: Bantam Books.

[B10] Cachia R. L. Anderson A. Moore D. W. (2016). Mindfulness, stress and well-being in parents of children with autism spectrum disorder: a systematic review. *J. Child Fam. Stud.* 25 1–14.

[B11] Carlson R. V. Boyd K. M. Webb D. J. (2004). The revision of the declaration of helsinki: past present and future. *Br. J. Clin. Pharmacol.* 57 695–713. 15151515 10.1111/j.1365-2125.2004.02103.x PMC1884510

[B12] Carona C. Moreira H. Silva N. (2016). Therapeutic applications of mindfulness in pediatric settings. *Adv. Psychiatr. Treat.* 22 16–24.

[B13] Center for Disease Control and Prevention [CDC]. (2018). *Autism Spectrum Disorder.* Available at http://www.cdc.gov/ncbddd/autism/index.html [accessed January 30, 2019].

[B14] Cohen J. A. S. Semple R. J. (2010). Mindful parenting: a call for research. *J. Child Fam. Stud.* 19 145–151.

[B15] Cohen S. Edwards J. R. (1989). “Personality characteristics as moderators of the relationship between stress and disorder,” in *Advances in the Investigation of Psychological Stress*, ed. Neufeld R. W. J. (Oxford, UK: Wiley), 235–283.

[B16] Cohen S. Kamarck T. Mermelstein R. (1983). A global measure of perceived stress. *J. Health Soc. Behav.* 24 385–396. 10.2307/2136404 6668417

[B17] Cohen S. Williamson G. (1988). “Psychological stress in a probability sample of the United States,” in *The Social Psychology of Health: Claremont Symposium on Applied Social Psychology*, eds Spacapan S. Oskamp S. (Newbury Park, CA: Sage).

[B18] Davis N. O. Carter A. S. (2008). Parenting stress and coping styles in mothers and fathers of toddlers with autism spectrum disorders: associations with child characteristics. *J. Autism Dev. Disord.* 38 1278–1291. 10.1007/s10803-007-0512-z 18240012

[B19] Davison G. (2000). Stepped care: doing more with less? *J. Consult. Clin. Psychol.* 68 580–585. 10.1037/0022-006X.68.4.580 10965633

[B20] de Kloet E. R. Joels M. Holsboer F. (2005). Stress and the brain: from adaptation to disease. *Nat. Rev. Neurosci.* 6 463–475. 10.1038/nrn1683 15891777

[B21] Donnchadha S. O. (2018). Stress in caregivers of individuals with intellectual or developmental disabilities: a systematic review of mindfulness-based research. *J. Appl. Res. Intel. Disabil.* 31 181–192. 10.1111/jar.12398 28833964

[B22] Duncan L. G. Coatsworth J. D. Greenberg M. T. (2009). A model of mindful parenting: implications for parent–child relationships and prevention research. *Clin. Child Fam. Psychol. Rev.* 12 255–270. 10.1007/s10567-009-0046-3 19412664 PMC2730447

[B23] Dykens E. M. Fisher M. H. Taylor J. L. Lambert W. Miodrag N. (2014). Reducing distress in mothers of children with autism and other disabilities: a randomized trial. *Pediatrics* 134 e454–e463. 10.1542/peds.2013-3164 25049350 PMC4187227

[B24] Estes A. Munson J. Dawson G. Koehler E. Zhou X. H. Abbott R. (2009). Parenting stress and psychological functioning among mothers of preschool children with autism and developmental delay. *Autism* 13 375–387. 10.1177/1362361309105658 19535467 PMC2965631

[B25] Estes A. Olson E. Sullivan K. Greenson J. Winter J. Dawson G. (2013). Parenting-related stress and psychological distress in mothers of toddlers with autism spectrum disorders. *Brain Dev.* 35 133–138. 10.1016/j.braindev.2012.10.004 23146332 PMC3552060

[B26] Faul F. Erdfelder E. Buchner A. Lang A. (2009). Statistical power analyses using G^∗^Power 3.1: Tests for correlation and regression analyses. *Behav. Res. Methods* 41 1149–1160. 10.3758/BRM.41.4.1149 19897823

[B27] Gilbert P. (2010). *Compassion Focused Therapy: The CBT Distinctive Features Series.* New York, NY: Routledge.

[B28] Griffith G. M. Hastings R. P. Nash S. Hill C. (2010). Using matched groups to explore child behavior problems and maternal well-being in children with down syndrome and autism. *J. Autism Dev. Disord.* 40 610–619. 10.1007/s10803-009-0906-1 19936904

[B29] Harnett P. H. Reid N. Loxton N. J. Lee N. (2016). The relationship between trait mindfulness, personality and psychological distress: a revised reinforcement sensitivity theory perspective. *Pers. Individ. Diff.* 99 100–105. 10.1016/j.paid.2016.04.085

[B30] Hartley S. L. Barker E. T. Seltzer M. M. Floyd F. Greenberg J. Orsmond G. (2010). The relative risk and timing of divorce in families of children with an autism spectrum disorder. *J. Fam. Psychol.* 24 449–457. 10.1037/a0019847 20731491 PMC2928572

[B31] Hayes S. C. Hofmann S. G. (2018). *Process-Based Cbt: The Science and Core Clinical Competencies of Cognitive Behavioral Therapy.* Oakland, CA: New Harbinger.

[B32] Hoffman S. G. Grossman P. Hinton D. E. (2013). Loving-kindness and compassion meditation: potential for psychological interventions. *Clin. Psychol. Rev.* 31 1126–1132. 10.1016/j.cpr.2011.07.003 21840289 PMC3176989

[B33] Hoffman S. G. Hayes S. C. (2018). The future of intervention science: process-based therapy. *Clin. Psychol. Sci.* 7 37–50. 10.1177/2167702618772296 30713811 PMC6350520

[B34] Hoge E. A. Bui E. Palitz S. A. Schwarz N. R. Owens M. E. Johnston J. M. (2018). The effect of mindfulness meditation training on biological acute stress responses in generalized anxiety disorder. *Psychiatr. Res.* 262 328–332. 10.1016/j.psychres.2017.01.006 28131433 PMC5526744

[B35] Hsiao Y.-J. (2018). Parental stress in families of children with disabilities. *Interv. school Clin.* 53 201–205. 10.1177/1053451217712956

[B36] Hwang Y.-S. Singh N. N. (2016). “Mindfulness,” in *Handbook of Evidence-Based Practices in Intellectual and Developmental Disabilities*, ed. Singh N. N. (New York, NY: Springer), 311–346.

[B37] Joels M. Baram T. Z. (2009). The neuro-symphony of stress. *Nat. Rev. Neurosci.* 10 459–466. 10.1038/nrn2632 19339973 PMC2844123

[B38] Kabat-Zinn J. (1990). *Full Catastrophe Living: Using The Wisdom of Your Body and Mind to Face Stress, Pain, and Illness.* New York, NY: Delacorte Press.

[B39] Lai W. W. Goh T. J. Oei T. P. Sung M. (2015). Coping and well-being in parents of children with autism spectrum disorders (ASD). *J. Autism Dev. Disord.* 45 2582–2593. 10.1007/s10803-015-2430-9 25800867

[B40] Lecavalier L. Leone S. Wiltz J. (2006). The impact of behaviour problems on caregiver stress in young people with autism spectrum disorders. *J. Intel. Disabil. Res.* 50 172–183. 10.1111/j.1365-2788.2005.00732.x 16430729

[B41] Ledford J. R. Gast D. L. (2018). *Single Case Research Methodology.* New York: Routledge 10.4324/9781315150666

[B42] Lovell B. Wetherell M. A. (2016). Behavior problems of children with ASD and perceived stress in their caregivers: The moderating role of trait emotional intelligence? *Res. Autism Spect. Disord.* 28 1–6. 10.1016/j.rasd.2016.05.002

[B43] Lunsky Y. Robinson S. Reid M. Palucka A. (2015). Development of a mindfulness-based coping with stress group for parents of adolescents and adults with developmental disabilities. *Mindfulness* 6 1335–1344. 10.1007/s12671-015-0404-9

[B44] Lupien S. J. McEwen B. S. Gunnar M. R. Heim C. (2009). Effects of stress throughout the lifespan on the brain, behaviour and cognition. *Nat. Rev. Neurosci.* 10 434–445. 10.1038/nrn2639 19401723

[B45] MacDonald A. (2016). “Staff training in Positive Behavior Support,” in *Handbook of Evidence-Based Practices in Intellectual and Developmental Disabilities*, ed. Singh N. N. (New York, NY: Springer), 443–466.

[B46] Maulik P. K. Mascarenhas M. N. Mathers C. D. Dua T. Saxena S. (2011). Prevalence of intellectual disability: a meta-analysis of population-based studies. *Res. Dev. Disabil.* 32 419–436. 10.1016/j.ridd.2010.12.018 21236634

[B47] McEwen B. S. (2012). The ever-changing brain: cellular and molecular mechanisms for the effects of stressful experiences. *Dev. Neurobiol.* 72 878–890. 10.1002/dneu.20968 21898852 PMC3248634

[B48] McIntyre L. L. Neece C. L. (2016). “Parent training,” in *Handbook of Evidence-Based Practices in Intellectual and Developmental Disabilities*, ed. Singh N. N. (New York, NY: Springer), 467–492.

[B49] McKenzie K. Milton M. Smith G. Ouellette-Kuntz H. (2016). Systematic review of the prevalence and incidence of intellectual disabilities: current trends and issues. *Curr. Dev. Disord. Rep.* 3 104–115. 10.1007/s40474-016-0085-7

[B50] Miodrag N. Hodapp R. M. (2010). Chronic stress and health among parents of children with intellectual and developmental disabilities. *Curr. Opin. Psychiatr.* 23 407–411. 10.1097/YCO.0b013e32833a8796 20592593

[B51] Morris K. R. Horner R. H. (2016). “Positive Behavior Support,” in *Handbook of Evidence-Based Practices in Intellectual and Developmental Disabilities*, ed. Singh N. N. (New York, NY: Springer), 415–441.

[B52] Mortensen J. A. Barnett M. A. (2015). Risk and protective factors, parenting stress, and harsh parenting in mexican origin mothers with toddlers. *Marr. Fam. Rev.* 51 1–21. 10.1080/01494929.2014.955937

[B53] Neece C. L. (2014). Mindfulness-Based Stress Reduction for parents of young children with developmental delays: implications for parental mental health and child behavior problems. *J. Appl. Res. Intel. Disabil.* 27 174–186. 10.1111/jar.12064 23813562

[B54] Newschaffer C. J. Croen L. A. Daniels J. Giarelli E. Grether J. K. Levy S. E. (2007). The epidemiology of autism spectrum disorders. *Ann. Rev. Public Health* 28 21.1–21.24. 10.1146/annurev.publhealth.28.021406.144007 17367287

[B55] Osborne L. A. Reed P. (2010). Stress and self-perceived parenting behaviors of parents of children with autistic spectrum conditions. *Res. Autism Spect. Disord.* 4 405–414. 10.1016/j.rasd.2009.10.011

[B56] Parker R. I. Hagan-Burke S. Vannest K. (2007). Percentage of all nonoverlapping data (PAND): An alternative to PND. *J. Spec. Educ.* 40 194–204. 10.1177/00224669070400040101

[B57] Parker R. I. Vannest K. (2009). An improved effect size for single-case research: nonoverlap of all pairs. *Behav. Ther.* 40 357–367. 10.1016/j.beth.2008.10.006 19892081

[B58] Podsakoff P. M. MacKenzie S. B. Lee J. Y. Podsakoff N. P. (2003). Common method biases in behavioral research: a critical review of the literature and recommended remedies. *J. Appl. Psychol.* 88 879–903. 10.1037/0021-9010.88.5.879 14516251

[B59] Reid C. Gill F. Gore N. Brady S. (2016). New ways of seeing and being: evaluating an acceptance and mindfulness group for parents of young people with intellectual disabilities who display challenging behavior. *J. Intel. Disabil.* 20 5–17. 10.1177/1744629515584868 25999396

[B60] Ruiz-Robledillo N. Sariñana-González P. Pérez-Blasco J. González-Bono E. Moya-Albiol L. (2015). A mindfulness-based program improves health in caregivers of people with autism spectrum disorder: a pilot study. *Mindfulness* 6 767–777. 10.1007/s12671-014-0316-0

[B61] Ruskin D. Campbell L. Stinson J. Sara A. K. (2013). Changes in parent psychological flexibility after a one-time mindfulness-based intervention for parents of adolescents with persistent pain conditions. *Children* 5:121. 10.3390/children5090121 30177644 PMC6162475

[B62] Shapiro S. L. Carlson L. E. Astin J. A. Freedman B. (2006). Mechanisms of mindfulness. *J. Clin. Psychol.* 62 373–386. 10.1002/jclp.20237 16385481

[B63] Shine R. Perry A. (2010). The relationship between parental stress and intervention outcome of children with autism. *J. Dev. Disabil.* 16 64–66.

[B64] Singh N. N. Lancioni G. E. Hwang Y.-S. Chan J. Shogren K. A. Wehmeyer M. L. (2017). “Mindfulness: An Application of Positive Psychology in Intellectual and Developmental Disabilities,” in *Handbook of Positive Psychology in Intellectual Disabilities*, eds Shogren K. A. Wehmeyer W. L. Singh N. N. (New York, NY: Springer), 65–79. 10.1007/978-3-319-59066-0_6

[B65] Singh N. N. Lancioni G. E. Karazsia B. T. Myers R. E. Winton A. S. W. Latham L. L. (2015). Effects of training staff in MBPBS on the use of physical restraints, staff stress and turnover, staff and peer injuries, and cost effectiveness in developmental disabilities. *Mindfulness* 6 926–937. 10.1007/s12671-014-0369-0

[B66] Singh N. N. Lancioni G. E. Karazsia B. T. Chan J. Winton A. S. W. (2016a). Effectiveness of caregiver training in mindfulness-based positive behavior support (MBPBS) vs. training-as-usual (TAU): a randomized controlled trial. *Front. Psychol.* 7:1549. 10.3389/fpsyg.2016.01549 27766088 PMC5053082

[B67] Singh N. N. Lancioni G. E. Karazsia B. T. Myers R. E. (2016b). Caregiver training in mindfulness-based positive behavior supports (MBPBS): effects on caregivers and adults with intellectual and developmental disabilities. *Front. Psychol.* 7:98. 10.3389/fpsyg.2016.00098 26903906 PMC4746712

[B68] Singh N. N. Lancioni G. E. Manikam R. Latham L. L. Jackman M. M. (2016c). “Mindfulness-based positive behavior support in intellectual and developmental disabilities,” in *Mindfulness in Positive Psychology: the Science of Meditation and Wellbeing*, eds Ivtzan I. Lomas T. (East Sussex: Taylor & Francis).

[B69] Singh N. N. Lancioni G. E. Medvedev O. N. Myers R. E. Chan J. McPherson C. L. (2018). Comparative effectiveness of caregiver training in mindfulness-based positive behavior support (MBPBS) and positive behavior support (PBS) in a randomized controlled trial. *Mindfulness* 1–13. 10.1007/s12671-018-0895-2 PMC7223775 32435317

[B70] Singh N. N. Lancioni G. E. Winton A. S. Karazsia B. T. Singh J. (2013). Mindfulness training for teachers changes the behavior of their preschool students. *Res. Hum. Dev.* 10 211–233. 10.1080/15427609.2013.818484

[B71] Singh N. N. Lancioni G. E. Winton A. S. W. Fisher B. C. Wahler R. G. McAleavey K. M. (2006). Mindful parenting decreases aggression, noncompliance and self-injury in children with autism. *J. Emot. Behav. Disord.* 14 169–177. 10.1177/10634266060140030401

[B72] Singh N. N. Lancioni G. E. Winton A. S. W. Karazsia B. T. Myers R. E. Latham L. L. (2014). Mindfulness-based positive behavior support (MBPBS) for mothers of adolescents with autism spectrum disorder: effects on adolescents’ behavior and parental stress. *Mindfulness* 5 646–657. 10.1007/s12671-014-0321-3

[B73] Singh N. N. Lancioni G. E. Winton A. S. W. Singh J. Curtis W. J. Wahler R. G. (2007). Mindful parenting decreases aggression and increases social behavior in children with developmental disabilities. *Behav. Modif.* 31 749–771. 10.1177/0145445507300924 17932234

[B74] Uusberg H. Uusberg A. Talpsep T. Paaver M. (2016). Mechanisms of mindfulness: the dynamics of affective adaptation during open monitoring. *Biol. Psychol.* 118 94–106. 10.1016/j.biopsycho.2016.05.004 27211913

[B75] Vago D. R. Silbersweig D. A. (2012). Self-awareness, self-regulation, and self-transcendence (S-ART): a framework for understanding the neurobiological mechanisms of mindfulness. *Front. Hum. Neurosci.* 6:296. 10.3389/fnhum.2012.00296 23112770 PMC3480633

[B76] Van Gordon W. Shonin E. Griffiths M. (2015). Towards a second generation of mindfulness-based interventions. *Aust. N. Z. J. Psychiatry* 49 591–592. 10.1177/0004867415577437 25801660

[B77] Wallace B. A. Shapiro S. L. (2006). Mental balance and wellbeing: building bridges between buddhism and western psychology. *Am. Psychol.* 61 690–701. 10.1037/0003-066X.61.7.690 17032069

[B78] Wallander J. L. Varni J. W. Babani L. Banis H. T. Wilcox K. T. (1989). Family resources as resistance factors for psychological maladjustment in chronically ill and handicapped children. *J. Pediatr. Psychol.* 14 157–173. 10.1093/jpepsy/14.2.157 2526867

[B79] Whittingham K. (2014). Parents of children with disabilities, mindfulness and acceptance: a review and a call for research. *Mindfulness* 5 704–709. 10.1007/s12671-013-0224-8

